# Household Characteristics, Residents’ Behaviors, and Seasonal Factors Associated With the Presence and Persistence of Aedes aegypti in a Dengue-Endemic Area

**DOI:** 10.7759/cureus.113423

**Published:** 2026-07-26

**Authors:** Alejandro Cortés-Meda, Guadalupe Ponciano Rodríguez, Guadalupe Silvia García-de la Torre, Julián Everardo García-Rejón, Jorge Fernando Méndez-Galván

**Affiliations:** 1 Department of Public Health, Faculty of Medicine, National Autonomous University of Mexico, Mexico City, MEX; 2 Department of Arbovirology Laboratory, Regional Research Center, Autonomous University of Yucatán, Merida, MEX; 3 Department of Research, Emerging Diseases Research Unit, Federico Gómez Children's Hospital of Mexico, Mexico City, MEX

**Keywords:** aedes aegypti, dengue, household, persistence, presence

## Abstract

Objective: This study aimed to evaluate the association between household characteristics, residents' preventive behaviors, seasonal factors, and the presence and persistence of *Aedes aegypti *in a dengue-endemic area. The findings are intended to identify modifiable household and behavioral factors that can inform targeted, seasonally adapted vector control strategies.

Methodology: This prospective cohort study was conducted in San Pedro Pochutla, Oaxaca, Mexico. A total of 68 inhabited dwellings were selected and characterized on a single occasion according to their structure, degree of marginalization, vulnerability, and environment. Over a period of 12 months, seven entomological evaluations were performed indoors and outdoors to assess the presence, persistence, and seasonal patterns of *Aedes aegypti*.

Results: A total of 476 visits were conducted, identifying 494 breeding sites positive for *Aedes aegypti *larvae or pupae. A total of 48.5% of the homes studied were positive on at least two occasions, with marked temporal variation, particularly during the rainy season (67.7%). Most breeding sites were located outdoors (96%), predominantly in controllable containers (73%). The most important habitats were drums, barrels/sinks/tubs/pools, cans/buckets, tires, and kitchen/washing utensils (72.8%). The characteristics of the dwelling that represented a risk for the presence and persistence of *Aedes aegypti* were the lack of mosquito nets on doors (OR=1.31, 95% CI=1.08-5.20), lack of pavilions on beds (OR=1.95, 95% CI=1.16-8.35), animals in the household (OR=2.27, 95% CI=1.19-7.63), and not applying insecticide indoors (OR=1.64, 95% CI=1.10-4.21).

Conclusion: Household conditions, human behavior, and seasonal variation significantly influence the presence and persistence of *Aedes aegypti*. These findings can inform targeted vector control strategies, particularly focusing on modifiable risk factors and seasonal interventions.

## Introduction

*Aedes aegypti *(*Ae. aegypti*) is considered the most important vector mosquito worldwide due to its role in transmitting multiple emerging arboviruses to humans, including dengue, Zika, chikungunya, yellow fever, and Mayaro virus. Among these, dengue is the most widespread globally [1-5]. This mosquito is found mainly in urban and suburban domestic environments, particularly in disadvantaged areas, where it finds optimal conditions for the development of its life cycle [6-9].

In Mexico, *Ae. aegypti* represents a major public health problem. It is estimated that nearly 60% of the national territory, where more than 50 million people live and where most of the agricultural, livestock, industrial, fishing, oil, and tourism centers of importance in our country are located, has conditions that favor the survival and distribution of this vector and the diseases it is capable of transmitting, with dengue the most important [10].

In 2023, Mexico reported the highest number of confirmed dengue cases to date, with 54,406 cases; of these, 25,570 (47%) corresponded to dengue with warning signs and severe dengue, with a case fatality rate of 0.79 per 100 cases [11]. The presence of *Ae. aegypti *has shown seasonal variations related to local ecology and meteorological parameters such as temperature, humidity, and rainfall [6,12].

Breeding sites of *Ae. aegypti *are predominantly found in domestic environments and are typically associated with artificial water-holding containers. These can be classified as follows: permanent or controllable (e.g., barrels, drums, cisterns, tanks, or buckets), which can be managed through larvicides and physical interventions; removable or disposable (e.g., tires, bottles, cans, and other containers); and natural (e.g., rock holes, coconut shells, tree hollows, and leaf axils). Any container capable of retaining water in areas where the mosquito is present may serve as a breeding site [2,10,13-24].

The life cycle of this mosquito is strongly influenced by household-level factors, such as inadequate water supply, lack of drainage systems, improper waste disposal, and poor housing conditions. In addition, a decreased perception of risk among the population negatively affects participation in vector control activities, such as the elimination of breeding sites, which is further aggravated by limitations in the financial resources allocated to vector control programs [25].

Currently, there is no widely effective antiviral treatment for dengue, and although vaccines exist, their use remains limited in many endemic settings. Therefore, prevention strategies rely primarily on reducing contact between humans and the vector [18,26]. Several studies have shown that the transmission of arboviruses is closely related to vector density, survival, and behavior [27-31].

Although socioeconomic status has been associated with the abundance of *Ae. aegypti *mosquitoes and dengue transmission, the probable driving factors that mediate this association, such as ecological factors specific to the household and understanding the spatiotemporal scale in which the encounter between mosquitoes and people occurs, have not been fully identified yet [32].

## Materials and methods

Methodology

**Figure 1:**
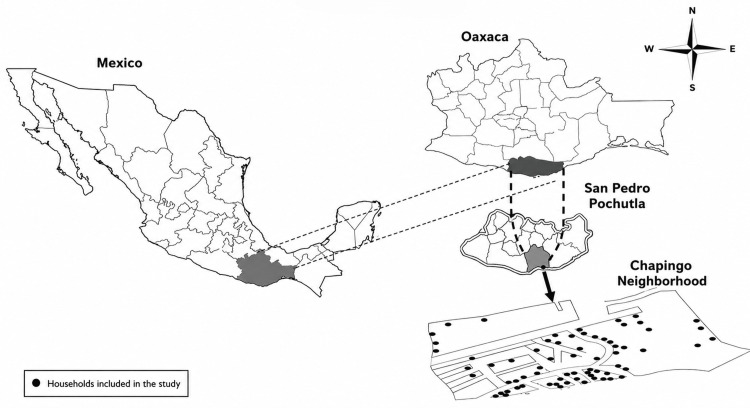
Place of the study. This image was created by the authors of this study using Microsoft PowerPoint (Redmond, WA: Microsoft Corp.) and Paint 3D (Redmond, WA: Microsoft Corp.).

This is a prospective cohort study carried out in inhabited households in a neighborhood of San Pedro Pochutla, Pochutla district, La Costa region, Oaxaca, between September 2020 and August 2021 (Figure 1) [33]. The sampling strategy was based on the recommendations of the entomological guide for studies in the larval and pupal phase developed by the National Center for Preventive Programs and Disease Control and previous studies, which recommend selecting 10-25% of the available households to achieve adequate entomological precision [32,34]. Of the 204 households in the study area, 86 (42.2%) were randomly selected. A total of 68 households (33.3% of all households and 79.1% of those initially selected) completed the seven scheduled entomological evaluations and were included in the final analysis. The households were chosen through a simple random selection process. If the father or mother of the family was not found or refused to participate, the next house located on the right was chosen.

Prior to the first entomological evaluation, and after obtaining written informed consent, a knowledge, attitudes, and practices (KAP) survey regarding dengue and *Ae. aegypti* was administered to the head of household of each participating dwelling. This baseline assessment aimed to characterize household-level behavioral factors potentially associated with vector presence.

Study site

San Pedro Pochutla is located between the 16°47’ north latitude and 96°28’ west longitude coordinates, at an altitude of 150 m above sea level. It is located approximately 230 km from the state capital. The climate is warm, subhumid with summer rains, average annual temperatures of 22-38°C, average humidity of 72% (64-80%), and average annual rainfall of 101 mm [35]. The population density of the municipality is 48.34 inhabitants/km^2^ . The housing conditions in the municipality are relevant because they show the need for basic services: nine (13%) do not have a connection to the public network for piped drinking water distribution, 42 (62%) depend mainly on the use of septic tanks, and only 26 (38%) are connected to the public network for waste disposal [35-37].

The study region is characterized by being hot and humid most of the year, with the dry period from January to May, November and December, and the rainy period from June to October (August and September being the months with the highest rainfall) [35]. This area is endemic to dengue, with an endemic profile that is usually seasonal, with large outbreaks from June to September, although cases can be observed at any time of the year. Circulation of serotypes one and two (dengue virus serotype 1 {DENV-1} and dengue virus serotype 2 {DENV-2}) has been observed, with a great predominance of the latter [38].

Entomological evaluations

Prior to the first entomological assessment, a mapping of the study area (Chapingo Neighborhood) was carried out (Figure 1). A total of seven entomological assessments were carried out inside and outside the homes under study as follows: three during the rainy season (September and October 2020 and August 2021) and four in the dry season (November 2020 and January, February, and March 2021).

For each of these months, the Stegomyia indices were calculated, which are essential for monitoring the *Ae. aegypti *population [6,39-41]. Those calculated in this project are the following: Positive House Index (PHI) = (households with positive containers/total households explored) × 100; Positive Container Index (PCI) = (positive containers/total containers with water explored) × 100; Breteau Index (BI) = (positive containers/explored houses) × 100.

The operational criteria for interpretation of PHI, PCI, and BI of the Pan American Health Organization (PAHO) and the methodological guide for entomological studies in the larval and pupal phase of the National Center for Preventive Programs and Disease Control (CENAPRECE) and other publications were followed (Table 1) [6,42].

**Table 1:** Interpretation of entomological indices. Own elaboration based on the methodological guide for entomological studies in the larval and pupal stages [34].

Operational control level	Positive Household Index	Positive Container Index	Breteau Index
Optimal	<1%	<0.5%	1-4.99
Good	1-2.99%	0.5-1.99%	5-9.9
Alarm	3-4.99%	2-4.99%	10-14.9
Emergency	≤5%	≤5%	≤15

The containers inside and outside each household were inspected for mosquito larvae and pupae and classified (controllable and disposable). All larvae (in the third and fourth stage) and pupae from the positive containers were collected using pipettes and scoops, counted, and recorded on forms unique to each household [43]. They were identified according to standard taxonomic keys [44].

Classification of breeding sites (breeding grounds)

They were classified as follows: controllable breeding grounds - these are containers that are present throughout the year, in use, and that due to their characteristics cannot be moved from the home. Disposable breeding grounds - all those containers that are not used, that are considered as garbage, and should be eliminated from the household, since, due to carelessness or the effect of rain, they retain water [40].

Data analysis

The data were analyzed using the statistical package Stata version 15 (College Station, TX: StataCorp LLC). The studied households and the behavior of their residents were characterized; the proportion of breeding sites positive for larvae and pupae was determined, as well as their location (inside or outside the homes), type (controllable or removable), and seasonal behavior. To improve the analysis, the households were classified according to their persistence for larvae and pupae as follows: persistent - households positive for *Ae. aegypti* breeding sites (larvae and pupae), for two or more months; non-persistent - households positive for *Ae. aegypti* breeding sites (larvae and pupae), for one month or never positive.

Persistence was operationally defined as household positivity in two or more visits. Although there is no universally accepted cutoff for defining household persistence of *Aedes aegypti*, this criterion was established a priori based on epidemiological considerations and the study's longitudinal design. Repeated detection of immature *Aedes aegypti* stages during independent household visits was considered indicative of recurrent household infestation rather than an isolated observation. This definition is consistent with previous longitudinal studies that conceptualize household persistence as the repeated presence of the mosquito over time rather than a single positive inspection [2].

Using the X^2^ test and Fisher's exact test, the association between the characteristics of the homes, KAP of the residents, and entomological indices with persistence of the mosquito *Ae. aegypti *was established. Finally, a multivariable logistic regression analysis was performed to evaluate the association between household characteristics and the presence and persistence of *Aedes aegypti*.

The multivariable logistic regression model was constructed using a forward selection procedure. Initially, variables showing a statistically significant association in the bivariate analysis (p<0.05) were considered. However, variable selection was not based solely on statistical significance. During model construction, the epidemiological relevance of the variables and the available evidence from the scientific literature were also considered to evaluate their potential role as confounding factors, even if they did not reach statistical significance in the bivariate analysis. Before fitting the model, potential multicollinearity among the independent variables was assessed using the variance inflation factor (VIF). All VIF values were below 5, indicating no evidence of relevant multicollinearity and supporting the simultaneous inclusion of the selected variables in the multivariable model. Model diagnostics also included an assessment of goodness of fit using the Hosmer-Lemeshow test. Finally, the most parsimonious model was selected, retaining only those variables that remained statistically significant and were epidemiologically consistent.

## Results

Seasonal and climatic factors at the study site

**Figure 2:**
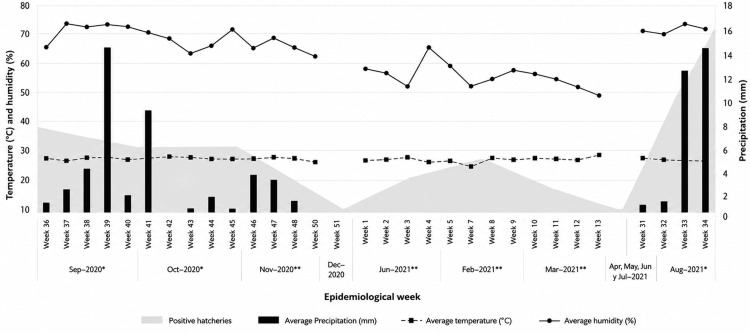
Average temperature, humidity, and precipitation per epidemiological week. *Rainy season. **Dry season. This image was created by the authors of this study using Microsoft PowerPoint (Redmond, WA: Microsoft Corp.) and Paint 3D (Redmond, WA: Microsoft Corp.). Temporal trends by epidemiological week, follow-up month, and season (rainy and dry) in average temperature (maximum and minimum), rainfall, humidity, and the total number of water containers harboring *Ae. aegypti* larvae and pupae in the Chapingo neighborhood of San Pedro Pochutla, Oaxaca.

During the studied period, an average annual temperature of 27.3°C, rainfall of 718 mm, and humidity of 64.2% were recorded (Figure 2).

Entomological indicators

**Figure 3:**
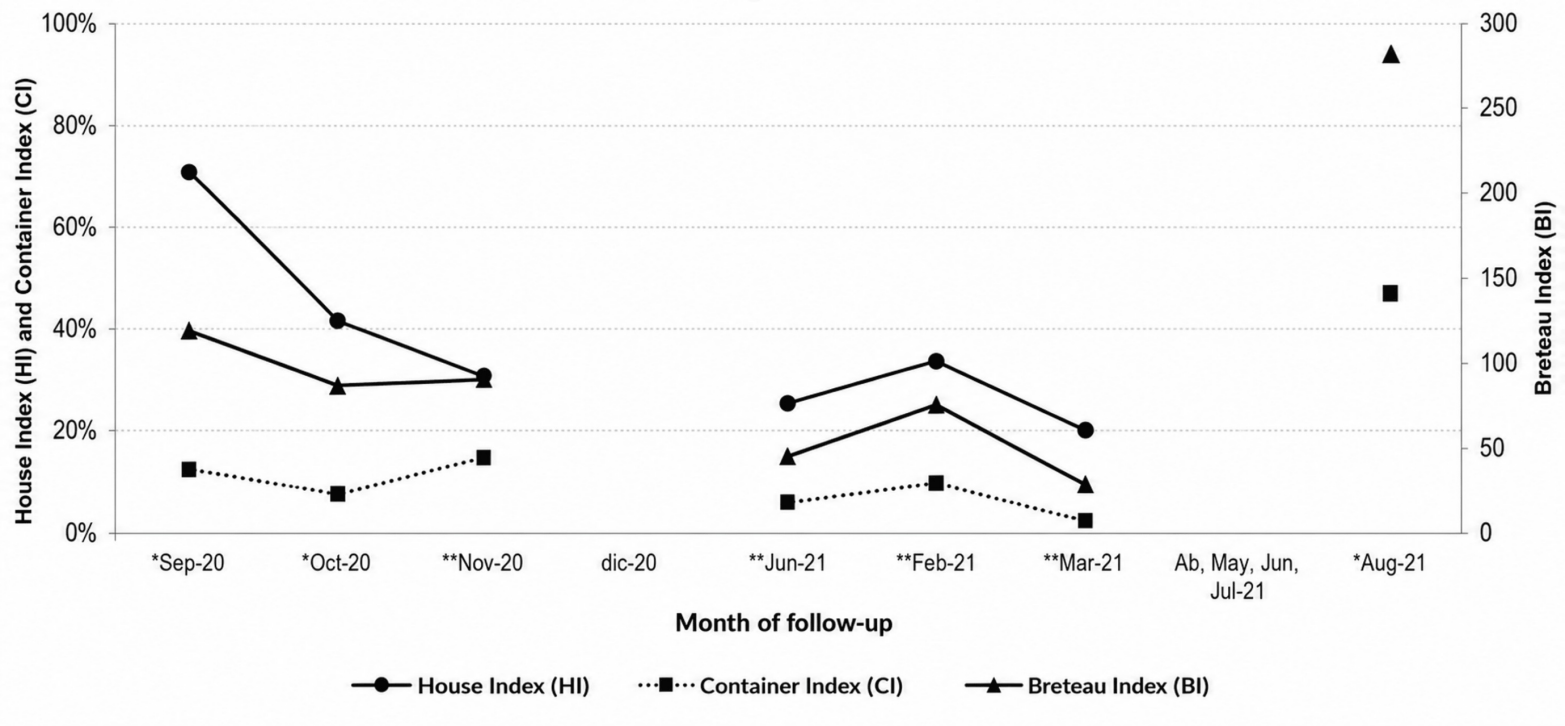
Monthly variation in the House Index (HI), Container Index (CI), and Breteau Index (BI) during the study follow-up period. *Rainy season. **Dry season. One-sample T-test. This image was created by the authors of this study using Microsoft PowerPoint (Redmond, WA: Microsoft Corp.) and Paint 3D (Redmond, WA: Microsoft Corp.).

As a result of the entomological evaluations, the House Index (HI), Container Index (CI), and Breteau Index (BI) were calculated to assess the levels of *Ae. aegypti* infestation in the study area (Figure 3, Table 2). Throughout the study period, the average values of all three indicators remained above the levels generally considered acceptable for vector control. The highest HI value was recorded in September 2020 (70.6%), whereas the highest CI and BI values were observed in August 2021 (46.8% and 280.9, respectively).

**Table 2:** Monthly values of the House Index (HI), Container Index (CI), and Breteau Index (BI) during the study period. *Rainy season. **Dry season.

Index	September-20*	October-20*	November-20**	June-21**	February-21**	March-21**	August-21*	Average
House Index (HI)	70.6%	41%	30.2%	25.0%	33.8%	20.6%	47.1%	38.4%
Container Index (CI)	12.9%	8.7%	15.2%	6.9%	10.6%	3.3%	46.8%	13.4%
Breteau Index (BI)	117.6	86.8	89.7	45.6	75	30.9	280.9	103.8

The overall average values obtained during the study period were 38.4% for the HI, 13.4% for the CI, and 103.8 for the BI. In general, HI and BI values were higher during the rainy season months (September and October 2020, and August 2021) than during the dry season months (November 2020, and February, March, and June 2021).

Notably, August 2021 showed the highest levels of entomological infestation, with the maximum values recorded for both the CI and BI. These findings indicate a greater proportion of positive containers and a higher level of household infestation during this period, demonstrating a high presence and persistence of *Ae. aegypti* in the study area.

Classification of positive containers

During the study, 14,484 breeding sites were found, of which 11,010 (76%) were negative since they did not contain water, 2,980 (20.6%) were negative with water, and 494 (3.4%) were positive for* Ae. aegypti* larvae and pupae. Of the 494 positive breeding sites, 474 (96%) were found in the peridomicile. Of these, 346 (73%) were controllable and 128 (27%) disposable.

When analyzing peridomicile breeding sites by season, 321 (67.7%) were found during the rainy season, of which 205 (63.8%) were controllable. Among these, barrels, kitchen and washing utensils, dishes, tanks/sinks/tubs/basins, and buckets or containers were the most frequent, representing 67.8% (139) of the controllable breeding sites. While the remaining 153 (32.3%) were identified in the dry season; of these, 141 (92.2%) were controllable, with barrels and tanks/sinks/tubs/basins being the most common, with 260 (54%). Of the disposable breeding sites, tires represented 57.8% (74) (Table 3). However, it is important to restate that, beyond the amount of rainfall, humidity had a more significant impact on the productivity of positive breeding sites.

**Table 3:** Positive hatcheries for Aedes aegypti larvae and pupae. Descriptive statistical analysis was performed using absolute frequencies “n” and percentages “(%).” No inferential statistical tests were applied to this table. C: controllable; D: disposable

Date	September 2020		October 2020		November 2020		June 2021		February 2021		March 2021		August 2021		Total, n (%)
Type of hatchery	n		n		n		n		n		n		n		
	C	D	C	D	C	D	C	D	C	D	C	D	C	D	
Exterior of the household															
Tank/deposits/basins/water stack	7	-	3	-	9	-	7	-	9	-	9	-	18	-	62 (13)
Barrels	21	-	7	-	9	-	6	-	20	-	7	-	27	-	97 (20.5)
Water tanks/cisterns/wells	4	-	4	-	2	-	5	-	7	-	3	-	3	-	28 (6.0)
Cans/buckets	6	1	2	-	3	2	4	-	4	1	2	-	13	20	58 (12.2)
Tires	2	13	-	30	1	1	1	-	3	-	-	-	7	30	88 (18.6)
Water dishes	4	-	1	-	7	-	-	-	1	-	-	-	-	-	13 (2.7)
Kitchen and washing utensils	1	1	1	-	2	-	-	-	-	-	-	-	33	2	40 (8.4)
Useless things (stuff)	-	5	1	-	5	1	4	-	-	-	-	-	2	-	18 (3.8)
Diverse small <5 L	-	8	-	1	-	7	-	-	1	-	-	-	5	3	25 (5.3)
Bottles/tins	-	1	1	-	3	-	-	-	-	-	-	-	16	-	21 (4.4)
Others	4	-	5	1	2	-	4	-	1	-	-	-	7	-	24 (5.0)
Exterior total	49	29	25	32	43	11	31	0	46	1	21	0	131	55	474 (100)

For the following analyses, it was decided to exclude natural and indoor breeding sites, since these together only represent 4% (20) of the total. Therefore, the total number of breeding sites with which the persistence of *Ae. aegypti* was measured decreased from 494 to 474.

Persistence

**Figure 4:**
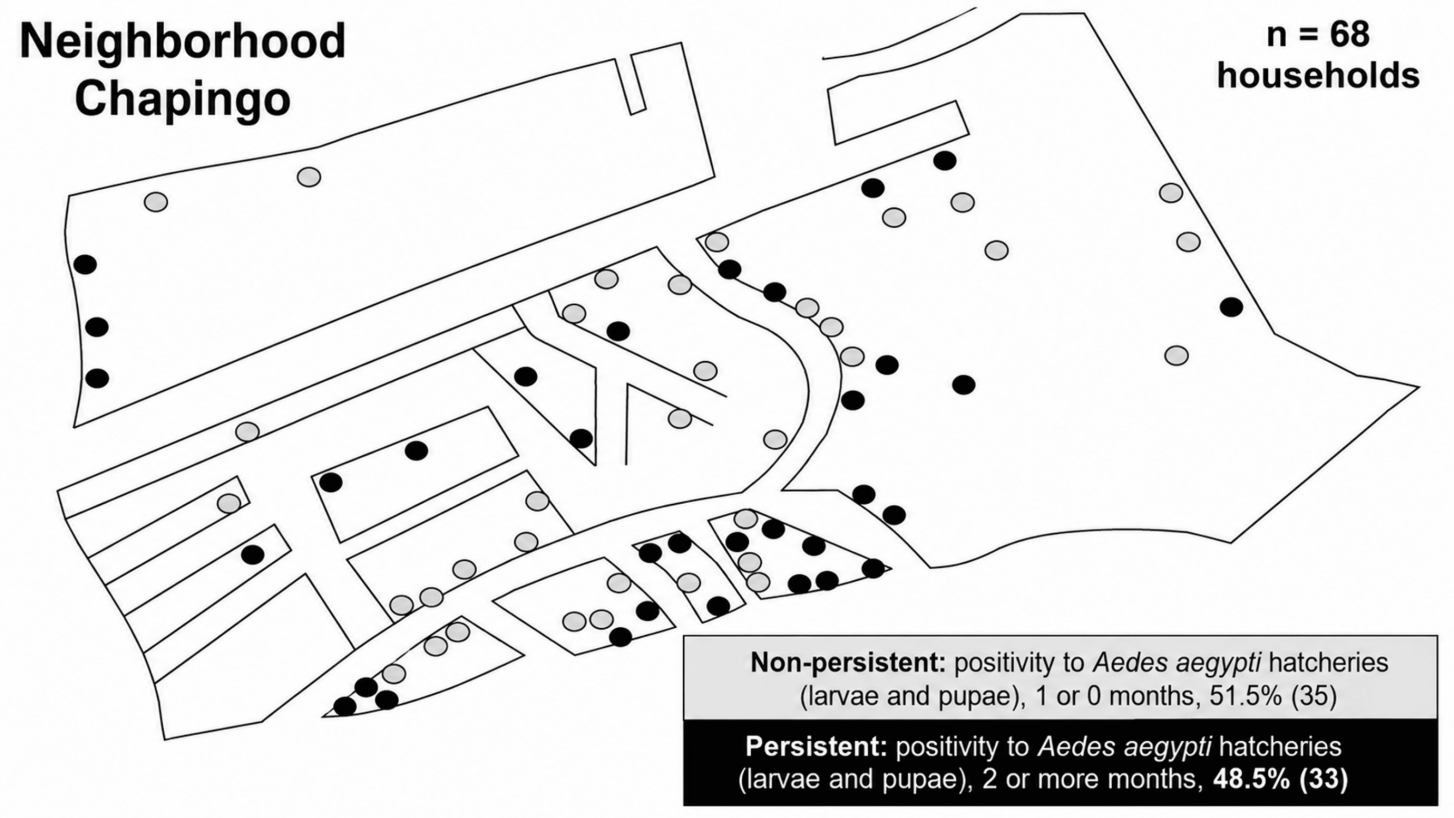
Spatial distribution of households with persistent and non-persistent Aedes aegypti positivity in the Chapingo neighborhood during the study period. This image was created by the authors of this study using Microsoft PowerPoint (Redmond, WA: Microsoft Corp.) and Paint 3D (Redmond, WA: Microsoft Corp.).

In total, 68 households accomplished a complete follow-up, of which 33 (48.5%) were considered persistent, since they tested positive two or more times throughout the study (Figure 4).

Demographic characteristics

The sociodemographic characteristics evaluated included sex, age, number of inhabitants per household, number of bedrooms per household, employment status, occupation, number of working hours per day, monthly income, housing tenure, social security coverage, distance between the household and the nearest health facility, and religion. Among the sociodemographic characteristics analyzed, the number of inhabitants per household, employment status, total family income, and social security coverage showed a significant association with the persistence of *Ae. aegypti* (Table 4).

**Table 4:** Association between demographic characteristics and household persistence of Aedes aegypti. Descriptive statistical analysis was performed using absolute frequencies (n) and percentages (%). Inferential statistical analysis was conducted using the chi-square (χ²) test or Fisher's exact test when expected cell frequencies were less than five. A p<0.05 was considered statistically significant. MXN: Mexican peso

Factor	Variables	Non-persistent, n (%)	Persistent, n (%)	Total, n (%)	X^2^	Fisher’s exact	p-Value
Inhabitants	1-3	16 (57.1)	12 (42.9)	28 (100)	-	6.71	0.001
	4-6	15 (46.9)	17 (53.1)	32 (100)			
	7 or more	4 (50.0)	4 (50.0)	8 (100)			
Employment situation	Employee	17 (56.7)	13 (43.3)	30 (100)	6.59	-	0.01
	Unemployed	16 (44.4)	20 (55.6)	36 (100)			
Family’s total income	<5000 MXN	21 (41.2)	30 (58.8)	51 (100)	-	7.92	0.005
	≥5000 MXN	12 (75.0)	4 (25.0)	16 (100)			
Social Security	Yes	17 (65.4)	9 (34.6)	26 (100)	7.76	-	0.005
	No	17 (41.5)	24 (58.5)	41 (100)			

Households with four to six inhabitants showed a higher proportion of persistence, with 17 (53.1%) households classified as persistent, compared with 12 (42.9%) households among those with one to three inhabitants. Likewise, households in which the head of household was unemployed showed a higher proportion of persistence, with 20 (55.6%) persistent households, compared with 13 (43.3%) among households in which the head of household was employed.

Similarly, persistence was more frequent among households without social security coverage, with 24 (58.5%) persistent households, compared with nine (34.6%) among households with social security coverage. In addition, households with a monthly family income below 5,000 Mexican pesos showed a higher proportion of persistence, with 30 (58.8%) persistent households, whereas households with a monthly income of 5,000 Mexican pesos or more showed only four (25.0%) persistent households (Table 4).

Housing and resident factors associated with the presence and persistence of *Ae. aegypti*

Various housing and resident characteristics were evaluated, including wall structure, vegetation surrounding the household, percentage of shade in the patio, ventilation, construction materials, kitchen characteristics, cooking fuel, the number of doors and windows leading outdoors, basic services, preventive practices, and animal ownership.

Variables significantly associated with the persistence of *Ae. aegypti* included the absence of mosquito screens on doors leading outdoors (Fisher’s exact test=2.38; p=0.02), absence of bed canopies or bed nets (χ²=2.59; p=0.04), participation in vector control campaigns (χ²=1.35; p=0.03), presence of animals inside the household (χ²=1.45; p=0.003), type of animal (χ²=2.06; p=0.02), absence of a corral or designated animal enclosure (χ²=3.64; p=0.02), number of exterior doors (χ²=1.99; p=0.04), and use of insecticide inside and outside the household (Fisher’s exact test=1.73; p=0.044 and Fisher’s exact test=1.71; p=0.025, respectively).

Households without mosquito screens on exterior doors showed 32 (54.2%) persistent households, compared with two (25.0%) households with mosquito screens on exterior doors. Likewise, persistence was observed in 23 (59.0%) households without bed canopies or bed nets, compared with 10 (35.7%) households that reported having them.

Persistence was also observed in 28 (50.9%) households with animals inside the household, compared with five (41.7%) households without animals. Among households with animals, persistence was more frequent in those with domestic animals, with 21 (56.8%) persistent households, compared with seven (38.9%) households with farm animals. Similarly, households without a corral or designated animal enclosure presented 20 (64.5%) persistent households, whereas eight (33.3%) persistent households were observed among households with a corral or enclosure.

Participation in vector control campaigns was associated with persistence, with 14 (53.8%) persistent households among those reporting participation, compared with 19 (46.3%) persistent households among those that did not participate. Regarding the number of exterior doors, persistence was observed in eight (38.1%) households with one exterior door, 11 (55.0%) households with two exterior doors, and 13 (59.1%) households with three or more exterior doors.

The use of insecticide inside and outside the household was also significantly associated with the persistence of *Ae. aegypti*. Persistence was observed in 30 (60.0%) households that did not apply insecticide indoors, compared with four (23.5%) households that reported indoor insecticide use. Similarly, persistence was observed in 30 (60.0%) households that did not apply insecticide outdoors, compared with four (23.5%) households that reported outdoor insecticide application.

On the other hand, drainage services, history of dengue within the household, presence of mosquito screens on windows, location of animal drinking containers, wall structure, flooring material, wall material, roof material, and number of windows to the exterior were not significantly associated with the persistence of *Ae. aegypti* (Table 5).

**Table 5:** Association between household characteristics and household persistence of Aedes aegypti. Descriptive statistical analysis was performed using absolute frequencies “n” and percentages “%.” Inferential statistical analysis was conducted using the chi-square (χ²) test and Fisher’s exact test. A p<0.05 was considered statistically significant.

Factor	Variables		Non-persistent, n (%)	Persistent, n (%)	Total, n (%)	X^2^	Fisher’s exact	p-Value
Drainage		Public sewer	6 (33.3)	12 (66.6)	18 (100)	2	-	0.08
		Septic tank	28 (57.1)	21 (42.9)	49 (100)			
History of dengue in the house		No	23 (46.9)	26 (53.1)	49 (100)	1.78	-	0.36
		Yes	11 (61.1)	7 (38.9)	18 (100)			
Mesh or mosquito screens on windows		No	27 (54.0)	23 (46.0)	50 (100)	1.43	-	0.08
		Yes	7 (41.2)	10 (58.8)	17 (100)			
Mesh or mosquito screens on doors		No	27 (45.8)	32 (54.2)	59 (100)	-	2.38	0.02
		Yes	6 (75.0)	2 (25.0)	8 (100)			
Pavilions on beds		No	16 (41.0)	23 (59.0)	39 (100)	2.59	-	0.04
		Yes	18 (64.3)	10 (35.7)	28 (100)			
Participation in vector control campaigns		No	22 (53.7)	19 (46.3)	41 (100)	1.35	-	0.03
		Yes	12 (46.2)	14 (53.8)	26 (100)			
Animals in the house		No	7 (58.3)	5 (41.7)	12 (100)	1.45	-	0.003
		Yes	27 (49.1)	28 (50.9)	55 (100)			
Type of animal		Domestic	16 (43.2)	21 (56.3)	37 (100)	2.06	-	0.02
		Farm	11 (61.1)	7 (38.9)	18 (100)			
Corral for animals		No	11 (35.5)	20 (64.5)	31 (100)	3.64	-	0.02
		Yes	16 (66.7)	8 (33.3)	24 (100)			
Location of animal drinking containers		Inside	2 (22.2)	7 (77.8)	9 (100)	-	3.5	0.13
		Out	14 (50.0)	14 (50.0)	28 (100)			
Wall structures		Continuous	20 (52.6)	18 (47.4)	38 (100)	1.19	-	0.72
		Discontinuous	14 (48.3)	15 (51.7)	29 (100)			
Flooring material		Cement	29 (56.9)	22 (43.1)	51 (100)	2.9	-	0.07
		Other materials	5 (31.2)	11 (68.8)	16 (100)			
Wall material		Bricks/blocks and other material	5 (38.5)	8 (61.5)	13 (100)	0.54	-	0.32
		Discarded material	29 (53.7)	25 (46.3)	54 (100)			
Roof material		Concrete	16 (50.0)	16 (50.0)	32 (100)	-	1.5	0.68
		Concrete/metal sheet	4 (40.0)	6 (60.0)	10 (100)			
		Others	14 (56.0)	11 (44.0)	25 (100)			
Exterior doors		1	13 (61.9)	8 (38.1)	21 (100)	1.99	-	0.04
		2	9 (45.0)	11 (55.0)	20 (100)			
		Three or more	9 (40.9)	13 (59.1)	22 (100)			
Windows to the exterior		1-2	13 (48.2)	14 (51.8)	27 (100)	1.02	-	0.13
		3-4	9 (52.9)	8 (47.1)	17 (100)			
		Five or more	10 (47.6)	11 (52.4)	21 (100)			
Use of insecticide inside the house		No	20 (40.0)	30 (60.0)	50 (100)	-	1.73	0.044
		Yes	13 (76.5)	4 (23.5)	17 (100)			
Use of insecticide to the outside of the house		No	20 (40.0)	30 (60.0)	50 (100)	-	1.71	0.025
		Yes	13 (76.5)	4 (23.5)	17 (100)			

Characteristics of the home and its inhabitants for the persistence of* Ae. aegypti*

Table 6 presents the results of the multivariable logistic regression analysis. The factors independently associated with the persistence of *Aedes aegypti* were the absence of mosquito screens on doors leading outdoors (OR=1.31; 95% CI: 1.08-5.20), the absence of bed nets or bed canopies (OR=1.95; 95% CI: 1.16-8.35), the presence of animals within the household (OR=4.27; 95% CI: 1.19-9.63), the presence of domestic animals (OR=2.01; 95% CI: 1.15-11.36), the absence of a corral or designated enclosure for animals (OR=2.46; 95% CI: 1.14-11.19), having two doors leading outdoors (OR=2.30; 95% CI: 1.22-10.71), and not applying insecticide inside the household (OR=1.64; 95% CI: 1.10-4.21).

**Table 6:** Multivariable logistic regression analysis of factors associated with household persistence of Aedes aegypti. Multivariable logistic regression analysis (n=68). All variance inflation factor (VIF) values <5. The Hosmer-Lemeshow test indicated an adequate model fit p=0.28. z: Wald z statistic; ref: reference categories were used for comparison.

Factor	Variables	OR	95% CI	Z-value	p-Value
Drainage	Public sewer (ref.)	-		-	0.448
	Septic tank	0.47	0.10-2.16	-0.96	
Mesh or mosquito screens on windows	No (ref.)	0.46	0.06-6.56	-0.65	0.601
	Yes	-		-	
Mesh or mosquito screens on doors	No	1.31	1.08-5.20	1.96	0.036
	Yes (ref.)	-		-	
Pavilions on beds	No	1.95	1.16-8.35	2.54	0.011
	Yes (ref.)	-		-	
Participation in campaigns	No (ref.)	-		-	0.167
	Yes	1.36	0.88-2.10	1.38	
Animals in the household	No (ref.)	-		-	0.006
	Yes	2.27	1.19-7.63	2.72	
Type of animal	Domestic	2.01	1.15-11.36	1.99	0.047
	Farm (ref.)	-		-	
Corral for animals	No	2.46	1.14-11.19	2.03	0.042
	Yes (ref.)	-		-	
Flooring material	Cement (ref.)	-		-	0.18
	Other material	2.88	0.61-13.62	1.34	
Exterior doors	1 (ref.)	-		-	-
	2	2.3	1.22-10.71	1.98	0.048
	3 or more	1.52	0.27-8.57	0.47	0.26
Use of insecticide inside the house	No	1.64	1.10-4.21	2.31	0.021
	Yes (ref.)	-		-	
Use of insecticide to the outside of the house	No	1.39	0.95-2.89	1.16	0.25
	Yes (ref.)	-		-	

These findings suggest that specific structural characteristics of the household, animal management practices, and indoor vector control measures play an important role in the persistence of *Aedes aegypti* in households in the Chapingo neighborhood of San Pedro Pochutla, Oaxaca. The model was adjusted for household size, monthly household income, and social security status; however, none of these variables showed a statistically significant association with the outcome.

## Discussion

*Ae. aegypti* is widely recognized as a domestic vector with a strong preference for human blood feeding, even in the presence of alternative hosts [1]. Its high competence for transmitting viral infections, particularly dengue, underscores its global public health importance.

This study identified key social, environmental, and behavioral factors associated with the presence and persistence of *Ae. aegypti*. The entomological methodology, based on the identification of positive breeding sites and the collection of larvae and pupae, proved effective for documenting vector presence, consistent with previous studies [23,45].

Environmental conditions, particularly seasonality, played a critical role in vector dynamics. A higher proportion of positive breeding sites was observed during the rainy season, with a total of 322 (68%), which is consistent with findings reported in previous studies [46,47]. High relative humidity, along with constant temperature ranges (23°-31°C), likely contributed to increased vector proliferation by accelerating development rates, shortening reproductive cycles, and enhancing survival [20,46-52].

Consistent with WHO recommendations, routine entomological surveillance remains essential to monitor fluctuations in vector density and geographic distribution, thereby informing risk assessment and control strategies [6]. In this context, the entomological indices used (PHI, PCI, and BI) demonstrated sensitivity to seasonal variations, with significantly higher values during the rainy season. Although these indices have been questioned due to their historical origin and the absence of well-defined dengue transmission thresholds [42,53,54], they remain useful for identifying priority intervention targets and understanding vector ecology [22,55].

The predominance of immature stages in the peridomiciliary environment aligns with previous reports [2,40]. Only 11 (2.4%) breeding sites were located indoors, likely due to frequent domestic use and cleaning of water containers, which disrupts larval development. Additionally, 48 (70%) households reported covering indoor water storage containers, further reducing indoor breeding potential [2,56].

The high reproductive activity in the home environment tells us about the adaptation of *Ae. aegypti* to indoor and outdoor habitats, which allows it to increase its reproduction opportunities and increase the rate of larval development [1,57]. This may have important implications for the transmission of dengue and other arboviruses.

According to other studies, most of the positive breeding sites are disposable, which was not found in this study, since of the total number of positive breeding sites identified, 346 (73%) were controllable [40,47,58]. The importance of controllable containers was maintained in both seasons; 303 (64%) of the total identified in the rainy season were of this type, while in the dry season it was 436 (92%). This requires that vector mitigation actions place greater interest in the habitats of larvae in the home environment [2].

The main positive breeding sites outside the houses during the rainy season were tires, barrels, buckets, and kitchen or washing implements. In the present work, the most relevant breeding sites were tanks/sinks, which represented 67% (318) of the total. Meanwhile, in the dry season, the most relevant breeding sites were barrels and tanks/tubs/sinks, which together represented 50%. Results that are comparable with other studies [14,18,23,59,60].

The high productivity of permanent containers may be due to the need to store water by the population for carrying out their daily activities, since the supply is irregular. In 65 (95%) households studied, water storage in large outdoor containers was reported. Its importance is due to the capacity to contain sufficiently large volumes of water for considerably longer periods than are suitable for complete larval development; therefore, they can be considered as key containers in both seasons [2,58]. Tires were the most important disposable breeding site throughout the study; their importance as a key breeder has been widely reported [2,16,47,58].

The high proportion of persistent households 33 (48.5%) may be explained by underlying socioeconomic and structural conditions, including inadequate water supply, poor sanitation, and suboptimal housing conditions. These factors have been consistently associated with increased vector proliferation [2,6-9].

Preventive measures were limited in the study population. A high percentage of households lacked mosquito nets on doors (59, 86%) and windows (51, 75%), and more than half did not use bed nets. Furthermore, 51 (75%) reported not using insecticides or repellents, and 41 (60%) had never participated in vector control campaigns. These findings suggest low risk perception and limited adoption of preventive behaviors, which may contribute to sustained transmission risk. The presence of domestic animals inside households may also facilitate vector persistence by providing additional blood sources and creating breeding opportunities through water containers such as troughs [2,15].

This study has limitations that should be considered when interpreting the findings. First, the relatively small sample size may have reduced the precision and stability of the estimates obtained from the multivariable logistic regression model. In addition, although the number of households lost to follow-up was limited, their exclusion may have affected the representativeness of the analytical sample and introduced potential selection bias. As with any observational study, the possibility of residual confounding cannot be completely excluded, despite adjustment for potential confounding variables in the multivariable analysis. Second, this study was conducted in a single dengue-endemic community, which may limit the generalizability of the findings to areas with different ecological, climatic, or socioeconomic characteristics. Third, household persistence was operationally defined as positivity in two or more visits. Although this definition was established a priori based on epidemiological considerations and the longitudinal design of the study, no universally accepted definition of household persistence currently exists, and alternative classification criteria may be used in future studies. Finally, repeated household visits may have influenced participants' behaviors over time, and temporary interruptions in data collection during the COVID-19 pandemic may have affected seasonal comparisons. Accordingly, the observed associations should be interpreted with caution and confirmed in prospective studies with larger sample sizes conducted in different epidemiological settings.

Despite these limitations, this study has several strengths. The prospective cohort design, repeated household evaluations over 12 months, and the integration of entomological, household, behavioral, and seasonal data enabled a comprehensive assessment of factors associated with the presence and persistence of *Aedes aegypti*. These findings may help inform targeted vector control strategies and provide a basis for future studies with larger sample sizes and broader geographic representation.

## Conclusions

This study from Mexico comprehensively characterizes the habitats of *Ae. aegypti *and shows that controllable household breeding sites are the main drivers of vector persistence in both rainy and dry seasons. In tropical endemic settings, relative humidity appears to be a key year-round environmental factor, even more stable than temperature and rainfall, favoring vector survival. Additionally, low household risk perception and free indoor access of domestic animals may increase domiciliary persistence. These findings support the development of a more focused entomological surveillance approach and provide evidence to guide context-specific prevention and vector control strategies that reduce human-mosquito contact in tropical endemic ecosystems.
